# Legacy of Death: How Colonial and Socio-Cultural Factors Support Capital Punishment in Indonesia

**DOI:** 10.1007/s10612-026-09886-z

**Published:** 2026-06-03

**Authors:** Lucrezia Rizzelli

**Affiliations:** Death Penalty Research Unit, Centre for Criminology, University of Oxford, 119 Frenchay Road, Oxford, OX2 6TE UK

## Abstract

The present article endeavours to analyse the roots and development of capital punishment in Indonesia, from its colonial past, through its independence, to the passing of the new criminal code in 2022. By focusing on history, political trends, religion and culture, through the lens of a postcolonial theoretical framework, it explains why the country has resisted the increased global appetite for abolition. Moreover, I posit that ‘coloniality’ plays a crucial role in the country’s efforts to maintain sovereignty over its internal policies, including human rights legislation, and its lack of permeability to the growing international pressures towards abolition. Capital punishment therefore emerges as a form of necropower, bestowing the state with the authority not only to kill, but to symbolically reclaim its right to act outside the moral economy presided by former colonisers.

## Introduction

If punishment is not merely the infliction of pain by a state, it requires justification (Berman, 2008). With over 700 people currently on death row in Indonesia, a thorough examination of capital punishment in this region becomes imperative (Amnesty International, 2023). This article aims to comprehensively explore the underlying factors contributing to Indonesia’s persistent resistance against the mounting global appetite for abolition, tracing how Indonesia’s criminal justice apparatus remains informed by inherited colonial rationalities. By following the historical trajectory of the death penalty in Indonesia, from its inception, through the colonial years and the independence war, to the passing of the new criminal code in 2022, this article sheds light on the enduring influence of the colonial legacy on existing legislation and its consequential impact on international dynamics, particularly within the realm of human rights discourse with the Global North.

Furthermore, this article examines other elements that have been crucial to the development and present retention of capital punishment for a variety of crimes, not least drug offences, which are not recognised under the International Covenant on Civil and Political Rights (ICCPR) as the ‘most serious crimes’; this puts Indonesia and some of its neighbours in Southeast Asia in breach of international law.

Initial scrutiny is directed towards the role of Islam in the conceptualisation of the death penalty as a possible form of punishment, as 81% of the Indonesian population is Muslim (Bureau of Statistics of the Republic of Indonesia, 2010). Secondly, I investigate the influence of Pancasila, a set of five tenets derived by Javanese religious traditions serving as the bedrock of the Indonesian state, on the relevant legislation (Iskandar, 2016). Finally, I discuss Indonesia’s positionality on the international stage, within an international human rights framework, underscoring the importance the Indonesian government places on sovereignty, especially in light of its colonial past. In doing so, I suggest that, like neighbouring Singapore, some of Indonesia’s resistance to human rights rhetoric and law is due to its entrenched position on its sovereignty over internal policy and legislation. The concluding section raises vital questions concerning foreign interference in the Indonesian legislative and political domain, highlighting the contentious role played by Northern (and former colonising) countries in influencing abolitionist narrative in Southern (and formerly colonised) jurisdictions. This exploration evidences the precarious nature of relationships with other influential neighbouring states when penal policies and practices are challenged, thereby revealing how power is negotiated through punishment.

This article draws from postcolonial theory and critical criminology to frame the persistence of capital punishment in Indonesia as part of a broader colonial legacy that extends beyond formal occupation. While much postcolonial scholarship has focused on colonialism’s spectacular violence, this paper considers how the juridical and bureaucratic logics of colonial rule persist in post-independence states, particularly through penal practices such as capital punishment (Comaroff & Comaroff, 2006; Mamdani, 2018). Through these lenses, colonial logics appear to persist not only through law, but also through the political rationales that legitimise state violence in the name of order and national identity.

## Capital Punishment as a Willing Legacy

While the death penalty had been present in the small kingdoms in the Nusantara region currently known as Indonesia even before the creation of the *Vereenigde Oost-Indische Compagnie* (VOC) and the subsequent colonisation by the Dutch, the practice had not been central to the administration of justice, nor codified as a systematic instrument of state power (ICJR, 2017; Reid, 1993). During the colonial era, however, it became a widely used tool to gain supremacy over the colonies and to silence dissent, resulting in an annual death toll equal to twice the number of capital sentences in Amsterdam (Bonke, 1999). In 1873, its practice was further strengthened by the issuance of the *Wetboek van Strafrecht voor Indonesie*, although it would be another 35 years before it would be enacted. It is noteworthy that even before the *Wetboek van Strafrecht voor Indonesie* made crimes such as theft and extortion, robbery and piracy punishable by death, capital punishment had been abolished in the Netherlands in 1870, following a decade of de-facto abolition (ICJR, 2017).

Sahetapy’s (1979) analysis of Dutch jurists’ justifications for the retention of the death penalty in the colonies suggests three main criteria: racial prejudice, public order, and the ingrained idea that the death penalty would not only incapacitate but deter criminals. Maintenance of public order, and therefore control of the territory, was likely the main practical justification for its retention, and indeed one jurist stated, ‘it would be irresponsible to relinquish a powerful tool such as the death penalty, which had a frightening nature that was not present in other punishment’ (ICJR, 2017, p. 46). Indeed, through the colonial history of the country, massacres and violence were used to ‘awe the population into obedience’ (p. 265) to deter guerrilla fighting and revolts against the colonisers, revealing the underpinning beliefs on deterrence (Luttikhuis & Moses, 2012). Regarding racial prejudice, Sahetapy (1979) argues that some jurists appeared convinced that the natives were prone to perjury, while others contended that they were simply more likely to believe in falsehoods, inadvertently perpetuating lies. While these beliefs were likely caused by poor translation from indigenous languages, and Dutch jurists’ lack of understanding of the local population’s values, the death penalty was nevertheless believed to be a harsh enough threat to discourage potential perjury or falsehood (Sahetapy, 1979).

A key turning point in Indonesian history was its independence war against the Netherlands (August 1945–December 1949) which after WWII had hoped to regain their colonial territories to recoup the debts accumulated during the conflict through the implementation of their development plan (Spruyt, 2005; Spector, 2007). This plan heavily relied on their former West Indies colonies and shaped the new approach to colonisation from violent expropriation to ‘transformative invasion’, based on resource development and winning over the population to convince them to willingly subject themselves to achieve modernisation. Indeed, the colonial army often co-opted locals to fight against their own people and the independence of their own country (Feichtinger & Malinowski, 2012). Despite the propaganda fed to the Dutch public on the humanitarian and peacekeeping aims of the army’s actions, reminiscent of the white-man’s burden that had justified centuries of colonisation and exploitation, the decolonisation period was not devoid of violence. While it is often idealised by Indonesians as their ‘heroic struggle against 350 years of brutal Dutch colonial oppression’ (Luttikhuis & Moses, 2012, p. 259), the line between genocide and warfare became blurred, and was worsened by the frequent inability of the Dutch army to distinguish between fighters and innocent civilians due to the flat organisational structure of the local resistance. Examples of what most Dutch scholarship defines as violent ‘excesses’ caused by the ‘force of the situation’ are the massacres in South Sulawesi in 1946–1957 where military officer Raymond Westerling killed thousands of natives and the extermination of the village of Rawagedeh in West Java in December 1947 (IJzereef, 1984).

While it might appear surprising that, post-independence, the Indonesian government decided to retain capital punishment, considering how it had been systematically used by the Dutch to oppress the population and had become a symbol of foreign sovereignty and racial discrimination (ICJR, 2017), Stoler’s (2008) concept of ‘imperial debris’ and Quijano’s (2000) ‘coloniality of power’ are demystifying. Through ‘imperial debris’, Stoler (2008) describes how colonial legacies are not static relics of the past, but enduring conditions that shape the present through institutional forms and governing rationalities. Indonesia’s continued use of capital punishment, embedded in criminal codes first drafted by Dutch colonial administrators, exemplifies such debris. Quijano’s (2000) ‘coloniality’, as well, survives colonialism, embedding racialised and hierarchical power relations into knowledge systems, institutions, and governance, explaining how Indonesia’s legal and penal systems continue to reproduce colonial logics by building new criminal codes on the bedrock of the *Wetboek van Strafrecht* and therefore perpetuating colonial legal structures. These frameworks suggest that colonial forms of governance do not merely linger but continue to actively shape the functioning of postcolonial states. As Quijano (2000) delineates the epistemic and institutional legacy of colonial rule, and Stoler (2008) highlights the debris that structures contemporary governance, Mbembe (2003) extends this argument by showing how sovereign power expresses itself in the right to kill, as it will be further explored in future sections. These perspectives coalesce in the Indonesian context, where capital punishment becomes a disciplinary tool, both inherited and reimagined to legitimise postcolonial authority.

## The Constitution and Drugs Legislation

While the death penalty is indeed a matter of controversy, the right to life has been enshrined in the Indonesian Constitution since 1945. Article 28 A states that ‘every person shall have the right to live and to defend his/her life and existence’ (Constitution of the Republic of Indonesia, 1945). This reasoning was used in 2011 to justify the conversion of Hanky Gunawan’s death sentence to 15 years in prison, with the Court also citing Article 3 of the Universal Declaration of Human Rights and Article 4 of Indonesia’s domestic Human Rights Law, also concerning the right to life (Butt, 2014). Additionally, article 28G(2) of the 1945 Constitution protects people from torture and inhumane or degrading treatment, while article 28B(2) states that individuals ‘shall have the right to protection from violence and discrimination’ (Constitution of the Republic of Indonesia, 1945). Yet, the Indonesian government constantly waives these rights through the imposition of death sentences for crimes that are internationally not viewed as deserving of capital punishment.

After a bloodily achieved independence, a push to create a uniquely Indonesian way of life emerged, mirrored by a brand-new Constitution—as well as new criminal code—that would create a line of demarcation from the colonial era (Munthe & Soge, 2018). However, as criminologists have long recognised how legal systems serve as instruments of social control (Garland, 1990; Hogg, Scott, & Sozzo, 2017), to establish command over a fragmented political landscape, President Sukarno made the unilateral decision to reinstate the *Wetboek van Strafrecht voor Indonesie* as the primary source of criminal law—this time named *Kitab Undang-undang Hukum Pidana (KUHP)*—through the enactment of Law No. 1 on The Regulation of The Criminal Law. Therefore, while there had been an appetite for a uniquely Indonesian criminal code, the Republic of Indonesia found itself regulated once again by Dutch legislation which had originated from the French Penal Code during the Napoleonic era, ultimately based on Roman law (Munthe & Soge, 2018). To this point, I believe relevant the introduction of Franz Fanon’s (1963) critique of post-independence regimes’ reproduction of colonial governance structures, and his warnings against postcolonial elites embracing coercive methods under the guise of sovereignty and national progress. Indonesia’s retention of colonial penal codes and rhetorical framing of capital punishment as an issue of sovereignty reflects this dynamic. Rather than signifying a clean break from colonialism, the continued use of capital punishment demonstrates a deeper entanglement with colonial modes of governance. Moreover, it raises the question of to what degree the justification of state-sponsored killing through the retention of capital punishment might have also offered a moral justification for the violent and repressive communist purges of the mid-1960s, which resulted in the death of 500.000 to one million people (Robinson, 2018; Melvin, 2018).

Through the years, there have been efforts to reform the criminal code, with the first draft having been developed in 1964, and the final draft being passed in December 2022, still retaining capital punishment (Najih, 2018; RUU KUHP, 2022). In summary, with Law No. 1, Sukarno passed up the opportunity to move the nation in a more humane direction by creating a line of continuity between the colonial past and the freshly independent Republic of Indonesia.

Since then, the constitutionality of capital punishment in the Indonesian context has been challenged on a variety of bases^1^ and rejected in almost every case. The only exception was the 1987 *Isto Sukarta bin Sapri* case, when the Supreme Court ruled against its constitutionality stating that it violated the Pancasila and its enshrinement of the right to life as a Preamble to the 1945 Constitution. However, this ruling did not have significant bearing on future cases since the Constitutional Court would only be established in 2003 (Supreme Court, 1987). As no challenge can be raised twice on the same grounds, the legal avenues towards abolition are scarce and unlikely to be successful (Coffey, 2015; Pascoe, 2015).

A further entrenchment of the death penalty within the legislation is represented by the multiple specialised laws prescribing such punishment for specific crimes. I will now focus on narcotic legislation, as drug offenders are disproportionately sentenced to death and executed. Starting from 1912, the use of opium was banned in the West Indies colonies by the Dutch colonisers, following the International Opium Convention, which represented the first international agreement aiming to superintend drug trades (Pietschmann, 2007). Then, right before the end of the Indonesian Independence war, the Dutch government emanated the 1949 Dangerous Substance Ordinance and on Prescription Drug Ordinance. In 1976, the Indonesian government ratified the 1961 Single Convention on Narcotics and the Draft Bill on Narcotics into law, making two drug crimes death-eligible (smuggling and dealing, except for cocaine and marijuana), in fear that drugs would be used as means for revolution. However, this law did not provide specific details as pertaining to psychotropic drugs, which lead to the issuance of Law No. 5 of 1997 on Psychotropics (ICJR, 2017).

Law No. 5 of 1997 was based on the same deterrence-based ideology previously used by the Dutch when dealing with the spread of opium users. It made available a judicially discretionary death penalty as maximum punishment for illegal traffickers, but not for users, who instead were meant to be rehabilitated (Leechaianan & Longmire, 2013). The same aim of eradication of illegal narcotics’ production, importation, exportation, and consumption continues to be present in the latest law pertaining to narcotics: Law No. 35 of 2009.

## Religion: Between Pancasila and Islam

As a preamble to the 1945 Constitution, President Sukarno established the Pancasila, a set of five principles, to represent the ‘fundamental State philosophy and Indonesian people’s worldview’ (Iskandar, 2016, p 725). His choice was driven by the idea of freedom of religion and ability to create unity through diversity, shying away from the majority Islamic religion, and instead basing the principles on what is commonly referred to as more ‘indigenous values’ (Iskandar, 2019). In fact, the Pancasila principles^2^ are rooted in the traditions of the kingdoms that used to be present in the Nusantara region since 400AC (Munthe & Soge, 2018).

Pancasila prioritises the common good over personal interests and decisions reached through consensus, aiming at reducing confrontation and increasing communal benefits. It is quite vague in its formulation, a characteristic likely due to its roots in Javanese aristocratic culture and the importance it affords to qualities such as self-restraint, and its view of ambiguity as an ‘elegant solution’ (p. 73) to maintaining a cultural balance and withholding judgement (Iskandar, 2019). Yet, this ambiguity opens the principles to different interpretations. The issues caused by this policy of ambiguity and dubiety are also compounded by the political belief that ‘the contemporary legal debates that have emerged in response to these conditions [have] aggravate[d] the problems rather than resolve[d] them’ (Bedner, 2013, p. 255), therefore creating a long-standing precedent of avoidance of critical legal issues, including capital punishment.

In conclusion, the Pancasila values represent the imperatives of what is intended as ‘Indonesia’s exceptionalism’. However, Pancasila was also used to legitimise the government’s authority, while at the same time preventing other principles or other belief systems from being considered as potential bases for the state foundation (Munthe & Soge, 2018). While they are generally regarded as positive by the people, they are also associated with the Sukarno presidency, denoted by policies aimed at power centralisation and authoritarianism; in this context, Pancasila can be seen as Sukarno’s compromise between the main post-independence actors—nationalists, communists, and Islamists—but also as a cover for his authoritarian measures. Following the end of his presidency, Pancasila’s association with that era has seen a decline in its popularity; however, in recent years, these principles have regained support, especially to counteract the desire of some Islamic political groups to replace it with sharia-based principles (Iskandar, 2016; 2019).

While Indonesia is not an Islamic State, 81% of its population is Muslim; yet, Islamic principles have struggled to find footing in the legal and political sphere due to the country’s pluralism and the population’s favourable view towards Pancasila (Bureau of Statistics of the Republic of Indonesia, 2010; Elson, 2010). In the 1955 elections, Muslim parties reported only 44% of voters’ support, and their hopes were further diminished by President Suharto’s push towards secularism and modernisation during the New Order era. After the fall of the Suharto regime, in 1998, there was a re-emergence of the more conservative and traditional Muslim groups; however this newfound fervour gained very little support, with only one fifth of Indonesians favouring an Islamic state, and a continuously declining support until an all-time low in 2009 (Elson, 2010). Despite the popularity of Pancasila and the lack of interest in Islamising the state, the government has resorted to a form of legal populism called ‘shariarization of the law’, which infiltrates values inspired by Islamic religion into laws that in turn often end up violating civil and political rights at the lower regional levels. This trend has also been mirrored by the increasing concessions made to Islamic parties in recent years on the premise that, ‘when [sharia-inspired regional moral regulations] have been tested in court, they have been upheld as legal because improving morality is clearly within the obligation of regional or municipal governments’ (Bowden, 2013, p. 156).

Overall, this penal populism, bolstered by media sensationalism and nationalistic and religious rhetorics, helps the state transform punishment, including executions, into performances of state legitimacy (Pratt, 2007). Colonial governance itself laid the groundwork for such populist spectacles, with public executions being used to instill fear, create social hierarchies, and establish political legitimacy among diverse populations. This practice resonates in the contemporary reliance on capital punishment as a visible assertion of state power. Penal populism in postcolonial Indonesia, then, is not a modern phenomenon but one shaped by colonial precedents of rule through spectacle and fear.

A wide range of Islamic groups and parties is present on the Indonesian territory, spanning the spectrum of religious conservatism and adherence to dogma, but there is an apparent agreement on support for capital punishment (Elson, 2010). According to mainstream interpretations of the *Qur’an*, the death penalty is an acceptable punishment for actions falling under the category of *hudad*, which are considered the worst of crimes, including apostasy, murder, and robbery. However, some scholars argue that a death sentence is not inevitable under sharia law. Bassiouni (2004) in fact reports that, ‘there is no mandatory death penalty, unless […] the *haraba*^3^ results in a homicide. In that case, they interpret the *Qu’ran’*s provision on *haraba* as requiring the death penalty’ (p. 180). The matter loses clarity when it comes to drug offences, for which sharia law does not specify a punishment, and therefore sanctions are justified not by a primary law in Islam but rather by juristic discretion and analogical reasoning (*qiyas*) (Mumīsa, Jaber, & Macalesher, 2015). The *Qur’an* however mentions *khamr*, a word with the literal meaning of ‘veil’, which refers to ‘any substance which causes clouding of the mind and interferes with rational thinking’ (Baasher, 1981, p. 240), and that historically has been interpreted as wine or alcohol. Through the years, Islamic jurists have applied analogical reasoning and expanded the concept of *khamr* to also include the different forms of drugs (Baasher, 1981; Mumīsa et al., 2015). While consuming drugs is considered a more serious violation than alcohol intoxication, in virtue of its potentially higher detrimental effects on its users, users’ families and wider communities, analogical reasoning requires that any punishment produced through it be similar or lesser than the one in the original law (flogging for *khamr*) (Syafi’i, 2009). It follows that punishing drug use with the death penalty may be beyond the scope of what is allowed by sharia law, therefore not respecting the criteria of exception to the right to life inscribed in the *Qur’an* (Mumīsa et al., 2015). However, according to some Islamic jurists, there exists provisions in *siyasah shar’iyyah* (governance according to sharia) to increase and even double the penalty of a crime as damage control, or to rebalance the stability of a community (Husin, 2016).

Despite common knowledge of Islam’s support for the death penalty, a much less discussed feature of the religion is the emphasis it puts on piety and forgiveness, as inscribed in the practice of *diya* (blood money), which is a pecuniary settlement between a perpetrator—or their family—and the family of a victim, to impose physical punishment or the death penalty instead of incarceration (Pascoe & Miao, 2017). According to the *Qur’an*, while it is ultimately up to the victim or the victim’s family whether to accept the *diya*, it is implied that it is an honourable and preferable option (Mumīsa et al., 2015).

This aspect of the religion however is often forgotten, with many countries with an Islamic majority relying on capital punishment to maintain social control, or purge society of the threats by outside agents. When in 1981 the Islamic Declaration of Rights was adopted, it read that, ‘human life is sacred and inviolable, and every effort shall be made to protect it. In particular no one shall be exposed to injury or death, except under the authority of the law’ (Azzam, 1998, p. 104). This effectively creates a loophole that allows for the death penalty and limits the right to life, moving further away from those ideals of forgiveness within the concept of *diya* and towards the punitive stance many Islamic states or states with a Muslim majority have embraced (Schabas, 2000). It is however worthwhile mentioning that, on the other end of the spectrum, there has been some limited effort from within Muslim communities to push for abolition; the Arab Institute for Human Rights and the organisation Hands Off Cain met in Tunis in 1995 with the support of the European Community, to discuss capital punishment. This meeting resulted in a call to all Arab states to ratify the Second Optional Protocol to the International Covenant on Civil and Political Rights on the premise that ‘within the Arab civilisational and cultural background, no real impediments exist and obstruct the evolution of secular legislations in the process of setting up limits to the death penalty and abolishing it’ (Schabas, 2000, p. 234). Indeed, for many Muslim states, capital punishment becomes ‘a policy choice, but not one which is necessarily mandated by the *Shari’a*. […] Muslim states can therefore curtail the death penalty by legislation and remain consistent with the *Shari’a*’ (Bassiouni, 2004, p. 185).

Lastly, it is to be noted that while the *Qur’an* allows for the death penalty, it is not the only holy book to provide such punishment. The *Bible* too condones and justifies capital punishment, and yet partly because of the Catholic Church’s public stance against the practice, and partly due to its tradition of separateness from the state, the teachings of the *Bible* related to the death penalty have been put aside in lieu of a more modern and secular approach to punishment (Anckar, 2014). A factor which might underpin the difference in religions’ stances on the death penalty might also be the conceptualisation of the individual; while in Protestantism there is an accent on individualism, Islam prioritises the *ummah* (community of believers), therefore giving it priority over the single individual (Anckar, 2014; Mathias, 2013). Therefore, the punishment of one person to guarantee the safety, or re-establish the morality, of the wider community is seen as acceptable. Contrarily, in societies with a majority of Protestants or Catholics this would be seen not only as an infringement of the individual’s right to life, but also as the ‘desecration of a sacred social entity’ (Mathias, 2013, p. 1255), a concept directly coming from the ‘cult of the individual’—or ‘religion of humanity’—as defined by Durkheim (1900) and deriving its roots from Humanism and the Enlightenment (Mathias, 2013). Beccaria (1778) offers a good exemplification of this concept, juxtaposing the illegality of taking one’s own life with the state’s ability to take an individual’s life:

Did anyone ever give to others the right of taking away his life? Is it possible that, in the smallest portions of the liberty of each, sacrificed to the good of the public, can be contained the greatest of all good, life? If it were so, how shall it be reconciled to the maxim which tells us, that a man has no right to kill himself, which he certainly must have, if he could give it away to another? (p. 104).

While sharia law is supposed to govern every aspect of life in Islamic societies, including the social order and criminal justice system, there exists the possibility for sharia laws to be amended or dismissed when necessary. This practice, called doctrine of necessity (*darura*), would allow for the death penalty to be outlawed in virtue of the evolving necessities of society. Yet, there appears to be an unwillingness among Islamic States, and within the Islamic-majority country of Indonesia, to consider the death penalty as obsolete and do away with it (Schabas, 2000). The role of Islam in Indonesia’s legal and moral traditions is further complicated by its framing as counter-colonial force, especially in opposition to Dutch and Western influence, despite how Islamic jurisprudence and governance have, at times, functioned as hegemonic systems that displaced or absorbed indigenous legal traditions not dissimilarly to standard colonial practices (Hefner, 2011). This does not mean to equate Islam with colonialism in the European sense, yet it invites a more nuanced view of how religious authority has operated alongside or in contrast to both *adat* (customary laws) and colonial systems. As Talal Asad (2003) argued, religious legal orders carry their own logics of discipline and normativity. In the Indonesian context, Islamic legal reasoning became one of several intertwined sources of legitimacy for capital punishment, complicating the simplistic binary framing between Western-colonial and indigenous-anti-colonial forces. This suggests that while Islamic legal reasoning is often viewed as opposing colonial rule, it has also contributed to the development of strict and disciplinary legal norms not too dissimilar from colonial frameworks. As a result, Indonesia’s postcolonial penal landscape can be seen as layered with multiple hegemonies, each shaping the moral and legal facets of punishment. It then becomes evident that abolition would have substantial repercussions at the political level, as the President would need to go against the will of the religious majority, an action that is furthermore hindered by the Indonesian’s tradition (based on *Pancasila*) to achieve change through consensus, often leading to stagnation and lack of discussion on major controversial practices or topics. This is further compounded by politicians’ fears of taking a stand and revealing their opinion on contentious issues (Iskandar, 2019).

## Sovereignty and International Relations

The second half of the twentieth century has seen an unprecedented cascade of states choosing to abolish the death penalty (Fijalkowski, 2001; Hood & Hoyle, 2015). Abolition has come about mostly in relation to two impulses: the need for reform of the state’s violent practices of punishment, and the beginning of the development of the international human rights of the individual (Mathias, 2013). Within the human rights framework, the death penalty can be described as ‘the ultimate denial of human rights. It is the premeditated and cold-blooded killing of a human being by the state. This cruel and inhuman and degrading punishment is done in the name of justice. It violates the right to life as proclaimed in the Universal Declaration of Human Rights’ (Amnesty International, 2007, p. 1).

In an effort to advance human rights, on February 23rd, 2006, Indonesia ratified the International Covenant on Civil and Political Rights (ICCPR), a treaty developed by the United Nations General Assembly in 1966, and entered into force in 1976, which provides a range of human rights, including freedom from torture and cruel, inhuman or degrading punishment (U.N. General Assembly, 1966). Despite its ratification of the ICCPR, theoretically aimed at fostering an environment favourable to abolition, Indonesia increased its execution regime in 2013 and has only marginally reviewed its capital punishment policies within the 2022 criminal code, therefore ignoring Article 6(6) on not delaying or preventing abolition (Primadianti & Zuhro, 2018). Many scholars have debated the ambiguous and permissive nature of the wording of the ICCPR, allowing retentionist countries to proceed undisturbed with their practices insofar as they claim to be following the criteria set out by the articles, without any independent external review body and without necessarily having to provide evidence of concrete steps towards abolition. This latest point is also compounded by the inability of the ICCPR to be legally binding, and the lack of consequences for countries that breach it, rendering it more aspirational than prescriptive (Primadianti & Zuhro, 2018). In summary, the lack of enforceable obligations in international law and the absence of a higher authority able to legally bind nations to international standards create the conditions whereby national powers can prevaricate when it is in their interest to do so (Azmi, 2015).

Unsurprisingly, during the General Assembly of 1982 on an abolitionist optional protocol that would then become the Second Optional Protocol to ICCPR, a coalition of states with Muslim majorities (Afghanistan, Iran, Iraq, Jordan, Kuwait, Lybia, Oman, Somalia and Sudan) opposed the resolution on the basis that capital punishment is allowed under sharia law (Schabas, 2000). The same justification was used in 1998, when during the Rome Diplomatic Conference, a group of Islamic States and nations from the Commonwealth of Caribbean opposed the exclusion of capital punishment from the *Rome Statute*, claiming that it was permissible under sharia law. Moreover, they accused Northern countries of wanting to force their criminal justice values on the rest of the world as a form of cultural imperialism, therefore shifting the discourse from a legal and human rights perspective to one centred on culture, religion, and national sovereignty. This shift creates a position in the debate aptly described by Schabas (2000) in the following terms: ‘the argument is disarming for many who oppose capital punishment in the “North”, and seductively demagogic for those who oppose it in the “South”’ (p. 234).

Article 6(2) limits the permissibility of the death penalty only for those offences defined as ‘most serious crimes’ (U.N. General Assembly, 1966). While international bodies seem to agree with the U.N. official definition of nothing ‘beyond intentional crimes with lethal and other extremely grave consequences’ set in the Safeguards Guaranteeing Protection of the Rights of Those Facing the Death Penalty (1984), what constitutes a ‘most serious crime’ seems to vary in different nations on a practical level, with many ICCPR ratifying states stretching this definition to fit societal or political views on what is ‘most serious’.

Drug offences, which are the most common charge amongst those executed in Indonesia, are contentious in the context of the ‘most serious crimes’ definition. While internationally they are strongly considered outside of the scope of the death penalty, as their production or sale is not carried out with the intention of causing lethal harm, there is a stronghold of retentionist countries—some of which are involved in ‘wars on drugs’—that defend their right to condemn drug offenders to death in light of the ‘state of emergency’ created by these substances (Human Rights Watch, 2008). Former Indonesian President Joko Widodo has often justified the presence of capital punishment for drug offences by sharing data collected by the National Narcotics Board (BNN) according to which between 40 and 50 Indonesian people die every day due to drugs, stretching the criteria of direct deadly injury to indirect effects of the substances. This data, on which President Widodo based his campaign against drugs, was found to be unsound in 2015, when a group of academics wrote an open letter published in the Lancet, highlighting the erroneous methodology and data collection, and subsequent skewed interpretation of the results (Irwanto et al., 2015, p. 250). Consequently, President Widodo scaled back the number of victims to approximately 20 to 30 each day but refused to address the broader issue on the fitness of drug crimes underneath the narrow umbrella of ‘most serious crimes’ (Kramer, 2017). This well exemplifies Brown’s (2009) argument of the adaptation of colonial forms by postcolonial penal systems under the guise of nationalism. Indeed, the persistence of capital punishment in Indonesia reveals an inherited logic of ‘legalised death’ as a spectacle of power, especially in its use against drug offences, often dramatised in public discourse, particularly when involving foreigners. These performances of punitive sovereignty in turn reinforce Indonesia’s autonomy in the face of global, especially Western, human rights pressures.

In response to the abolitionist wave of the late 1990s, retentionist countries have become more vocal about their support for capital punishment, actively pushing back during debates on abolition and on the establishment of a moratorium on two premises: the additional further international pressures to conform to the now predominant abolitionist movement; and the imposition of Northern imperialistic values over countries that, for the vast majority, used to be colonised territories (Schabas, 2000).

Indonesia has consistently voted against the worldwide moratorium proposed by the UN,^4^ except for the General Assembly of 2012, when it abstained from voting. While that might have been perceived as a glimmer of hope for abolitionists, when news of the abstention became public, president Yudhoyono reacted by resuming executions after a four-year de-facto moratorium between 2009 and 2012 (Coffey, 2015; McRae, 2017). Despite the contrary votes to the moratorium and the ambiguous abstention, executions have ceased in Indonesia since 2016 (Amnesty International, 2023). This erratic use of the death penalty highlights how despite the claims that it is necessary for social order and deterrence, it serves a political function, as portrayed by the clustering of executions during election times, when it becomes an instrument for public support by showcasing a ‘tough on crime’ attitude (Coffey, 2015). The absence of executions since 2016 has also been justified by the government through the need to reform the death penalty legislation, conceptualising the practice in a new ‘Indonesian way’. The closest description of what it means to do capital punishment the ‘Indonesian way’ can be found in the 2022 criminal code, through which the death penalty has become an alternative punishment, with the possibility of individuals being sanctioned to a probationary death sentence^5^ (RUU KUHP, 2022).

The importance of international relations in the death penalty debate is multifaceted. Firstly, the technological advancements of the twenty-first century have bridged geographical distances, raising awareness of human rights violations and questionable practices the world over. Secondly, due to the global economy and the delicately balanced pecuniary relationships between countries, national sovereignty is faced with international interests directly connected to the local economy (Galineau, 2015). While the death penalty per se may not automatically be a matter of foreign policy, as the latter is defined as ‘the strategy or approach chosen by the national government to achieve its goals in its relations with external entities’ (p. 13), it becomes one when foreign nationals are sentenced to death (Azmi, 2015).

The Indonesian government has shown contradicting stances on the matter of international relations and sovereignty. It has refused to grant clemency to foreign nationals condemned to death in Indonesia when their country of origin has asked the government to exercise clemency, making a point of asserting its sovereignty on these decisions. Yet, it has also gone to great lengths to save the lives of its citizens sentenced to death abroad, like in the case of Indonesian workers in Saudia Arabia, with the payment of *diya*, and the investment in legal representation (Galineau, 2015; Novak, 2014). This resistance to outside pressures on national policy reflects Spivak’s (1999) arguments that postcolonial states negotiate international legal regimes through ‘strategic essentialism’, asserting cultural difference and legal independence to shield themselves from external normative demands. Mbembe’s (2003) theory of necropolitics is also a helpful reminder of how states exercise sovereignty through their capacity to decide who may live and who must die, and while Mbembe focuses on exceptional violence in the context of occupation and war, his framework can be extended to the postcolonial penal domain. The picture emerging in Indonesia, in light of the government’s perceived necessity to extend its necropower to the international stage, sees capital punishment function as a site where sovereignty is not only asserted, but symbolically reclaimed from former colonisers and contemporary moral imperialists (Kauzlarich et al., 2001). In practice, this is exemplified by how the Indonesian government has often failed to respect international agreements guaranteeing safeguards for foreign nationals,^6^ as well as by the disproportionate number of foreign nationals executed during the Widodo administration (Amnesty International, 2015). This both strengthened the government’s narrative of drug offenders being outsider corruptors of Indonesia, and raised issues of arbitrariness and discrimination, as foreign nationality has become a risk factor for execution, once again reproducing forms of subaltern violence drawn on the basis of racial and cultural lines (ICJR, 2019). Indeed, by excluding from its retentionist discourse the voices of those who are most vulnerable to being subjected to capital punishment (poor, often rural individuals, or foreign defendants), capital punishment becomes a disciplining mechanism that in turns reproduces subaltern silence akin to what Spivak (1988) wondered with his question ‘Can the subaltern speak?’, further reinforcing governmental control over justice narratives.

In 2012, Babcock pointed out that ‘for the first time in recent history, retentionist states are limiting the scope of the death penalty in direct response to concerns about reciprocal action by other retentionist states’; however, Indonesia seems to be unaffected by this trend and unconcerned with the mixed signals sent to international parties about its stance on capital punishment and foreign nationals. Moreover, through the decades since its independence, the Indonesian state has itself engaged in its own internal imperial logics, particularly in West Papua, with its patterns of territorial domination, legal assimilation, demographic restructuring, and violent suppression of dissent (Gietzelt, 1989; Druce, 2020). The incorporation of West Papua into the Republic of Indonesia through the 1969 ‘Act of Free Choice’, has been accompanied by a project of Indonesianisation, which included the imposition of Bahasa Indonesia, Pancasila ideology, and Javanese cultural norms over a population that shared very tenuous ties with the historic, religious, and linguistic features of the rest of Indonesia (Gietzelt, 1989). Furthermore, transmigration programmes supported by the World Bank transformed the demographic makeup of West Papua, further marginalising indigenous people (Druce, 2020). This internal colonial process operates under a similar logic that Indonesia resists from abroad: the assumption that a foreign government may define the boundaries of the national self, impose its moral and legal order, and use force to maintain its territorial unity while exploiting the land’s resources. Indeed, many of the administrative tools used in West Papua, such as military occupation, resource extractions, and the criminalisation of dissent, leading often to violent suppression of independent aspirations, resemble the very colonial practices Indonesia critiques when defending its sovereign right to execute foreign nationals or retain harsh penal codes (Viartasiwi, 2018).

In conclusion, sovereignty appears to be at the centre of the death penalty political debate at the international level. In response to the executions of foreign nationals in Indonesia, France and the UK have expressed open condemnation, Brazil has withdrawn its ambassador, while Australia has condemned the government’s choice to proceed with the executions notwithstanding the substantial financial aid they had provided to Indonesia after the 2004 tsunami; yet, many countries are not as loud in their condemnation of this practice in the Indonesian context in fear that their own foreign nationals might be fast-tracked to the next round of executions (Azmi 2015; Galineau, 2015).

As Andrew Carr stated, ‘for a diplomat, the death penalty cases are always the hardest ones because they involve a supreme act of sovereignty—the foreign state believing it has the right to take the life of someone that’s committed a crime—but also a supreme loss of sovereignty that a country isn’t able to protect its citizen overseas’ (in Galineau, 2015, p. 1).

## Conclusion

I have presented an overview of the historical, legal, religious, and cultural contexts that explain, at least partially, why Indonesia retains capital punishment. Despite its efforts to distance itself from its colonial past, legislation regarding capital punishment, a practice heralded by the Dutch colonisers, remains present in the criminal code 80 years after Indonesia’s independence.

Abolitionist influences from Northern countries encounter significant resistance in Indonesia. As this article has argued, the death penalty operates as more than a remnant of colonial legislation; it is an active site through which sovereignty, moral authority, and national identity are performed in the postcolonial state. Embodying ‘imperial debris’, capital punishment in Indonesia is not an inert juridical ruin, but rather an inheritance that has been repurposed to shape the moral and political framework upholding the state’s power both nationally and internationally.

Since its independence, Indonesia has witnessed the succession of several eras, characterised by vastly different approaches to governance and tolerance towards political diversity. These range from the purge of communism in the mid-1960s, resulting in the death of 500.000 to one million people, through the authoritarian New Order, to the current Reformasi era which has seen a certain Westernisation of the country. Nevertheless, Indonesia takes pride in maintaining its South-East Asian values and what it defines as ‘Indonesian exceptionalism’, openly critiquing both the communist approach of other Asian countries and the more capitalistic and individualistic one prevalent in the West, despite actively participating in the global capitalist economy. Indonesia is therefore left grappling with an uncomfortable positionality, having to adhere to capitalistic rules to support national economic growth, which often clash with its core values, and often engaging in relationships of economic servitude or exploitation with its former coloniser and other colonising forces in the region, while at once engaging in its own imperial logics in West Papua. It then becomes apparent that claims of national sovereignty on the legislative arena become one of the few strongholds the Indonesian government can cling to without fearing severe economic repercussions. The death penalty thus emerges as a form of necropower, with the state’s authority to kill not only exercised over the individual, often of foreign nationality, but also symbolically reclaiming its right to act outside of the moral economy presided by former colonisers. This dynamic is further strengthened by the narrative the government has adopted when describing the ‘narcotics emergency’, that of foreign drug offenders coming to Indonesia to weaken the new generation. While it distances the local governance from a problem that is indeed endemic, as well as from its failure to decrease offending rates through punitive deterrent strategies, it also challenges foreign imperialism. By pointing to outsiders as the importers of rotten values and damaging substances, the high rates of death sentences amongst foreign nationals charged with drug offences and the disproportionate number of foreigners executed function as a paradoxical reiteration of colonial modes of control under the guise of sovereign resistance. Indeed, this assertion of sovereignty as opposition to the echoes of colonialism is complicated by the Indonesian state’s mimicry of colonial practices, first and foremost its retention of a penalty that is embedded in colonial codes. Capital punishment thus becomes in itself an echo of colonial domination, entrenching what it purports to reject.

In an era where international relations increasingly recognise the harms of the past and the ethical issues surrounding the blind exportation of Northern values and legislation to the Global South, even persuasive abolitionist narratives are scrutinised not only through an economic lens, but also through historical ones. Cultural imperialism is therefore rejected and opposed through the retention of capital punishment, which embodies both a perceived necessity for a country which has endured significant political instability, and a form of resistance against the Global North.

## Footnotes

1 In 2007, the Supreme Court ruled against a challenge on the constitutionality of the death penalty for drug offences, stating that they belong to the ‘most serious crime’ category, due to their impact on the population and their potential to weaken the country’s youth and values (Sianturi v State, 2007). In 2008, the Supreme Court upheld the constitutionality of the method of execution (firing squad), claiming that some pain is unavoidable, and therefore does not amount to torture (Nurhasyim v State, 2008). In 2012, the Supreme Court ruled that violent robberies resulting in serious injuries or death are also among the ‘most-serious crimes’, and thus death-eligible (Raja Syahrial v State, 2012).

2 The first principle of Pancasila is the God-head value, enshrining the role of religious values in the shaping of the country and guaranteeing tolerance of different religions. The second value of Humanity instead postulates that individuals need to respect themselves and others in virtue of their human nature, with the implication that laws are meant to protect people and not to harm them. Unity represents diversity and pluralism, and how they can be a base for Indonesian nationalism, while the Popular value stresses the democratic nature of the country, and specifically how crucial consensus through representative democratic deliberation is. Lastly, the principle of social justice is conceptualised as the natural goal of the previous values, all concurring to the creation of a fair and just social system (Najih, 2018).

3 Brigandage, or going to war against Allah and the Prophet or any Islamic ruler (Bassiouni, 2004).

4 For UN resolutions, see: https://digitallibrary.un.org/search?ln=en&p=Moratorium%20on%20the%20use%20of%20the%20death%20penalty%20%3A%20resolution%20%2F%20adopted%20by%20the%20General%20Assembly&f=&c=Resource%20Type&c=UN%20Bodies&sf=&so=d&rg=50&fti=0.

5 Conversion to life imprisonment by Presidential Decree if it is not carried out within ten years, given good conduct and lack of negative impact on the public (RUU KUHP, 2022).

6 Some examples are: not informing embassies when foreign nationals were sentenced to death or about to be executed, not giving foreign nationals and their representatives a 72 hour notice prior to the execution and not offering interpreters at every stage of the investigative and legal proceedings, all of which they should do according to the Vienna Convention on Consular Relations of 1963 (Amnesty International, 2015).
